# Acceptance and Commitment Therapy Group Intervention for Parents of Children with Disabilities (Navigator ACT): An Open Feasibility Trial

**DOI:** 10.1007/s10803-022-05490-6

**Published:** 2022-03-03

**Authors:** T. Holmberg Bergman, E. Renhorn, B. Berg, P. Lappalainen, A. Ghaderi, T. Hirvikoski

**Affiliations:** 1 Department of Women’s and Children’s Health, Pediatric Neuropsychiatry Unit, Center for Neurodevelopmental Disorders at Karolinska Institutet (KIND), Gävlegatan 22B, 11330 Stockholm, Sweden; 2 Habilitation and Health, Region Stockholm, Stockholm, Sweden; 3 Center for Psychiatry Research, Stockholm, Sweden; 4 Department of Clinical Neuroscience, Karolinska Institutet, Nobels väg 9, 17165 Stockholm, Sweden; 5 Department of Psychology, University of Jyväskylä, PO Box 35, 40014 Jyväskylä, Finland

**Keywords:** Parental stress, Parental distress, Autism spectrum disorder, Disability, Treatment, Feasibility

## Abstract

Parents of children with autism spectrum disorder and other disabilities report high levels of distress, but systematically evaluated interventions are few. This study aimed to evaluate the feasibility of a novel, manualized Acceptance and Commitment Therapy group intervention (*Navigator ACT*) in a sample of 94 parents of children with disabilities. Feasibility was measured by treatment completion, credibility, and satisfaction, and preliminary outcomes by using self-rating scales administered at the baseline, post-intervention, and follow-up. The results imply the intervention is feasible in the context of Swedish outpatient habilitation services. A preliminary analysis of the outcome measures suggests that parents experienced significant improvements in well-being. The results indicate that the treatment is feasible and should be evaluated in a randomized controlled trial.

Parenting a child with a disability poses many challenges (Lindo et al., 2016) and can lead to extreme parental stress and mental health issues. Research findings over several decades confirm that parents of children with disabilities, such as Autism Spectrum Disorder (ASD), cerebral palsy (CP), intellectual disability (ID), acquired brain injury, Attention Deficit Hyperactivity Disorder (ADHD), and chronic conditions experience high stress, physical concerns, and distress, including depression and anxiety (Hayes & Watson, 2013; Keenan et al., 2016; Scherer et al., 2019; Theule et al., 2013). Parental stress is defined as stress arising from an imbalance between the demands (stressors) of being a parent and the internal and external resources available to meet these demands (Deater‐Deckard, 1998). Distress is a concept that is similar to stress but refers to emotional states such as sorrow, pain, depression, and anxiety that result from failing to adapt to stressors (Fink, 2016). Caregivers appear to share many similarities in terms of stress and distress, regardless of the child’s specific disability. Commonly mentioned causes include the burden of care, the child’s comorbid emotional and behavioral problems, worry for the child’s future, stigma, and the parent’s own internal judgments—e.g. failing to live up to one’s own or others’ expectations of being an “ideal parent” (Barroso et al., 2018; Broberg, 2011; Weitlauf et al., 2014). Furthermore, lack of social support (Lindo et al., 2016), ableism, and social prejudice against disabilities can cause parents to feel stress. As a response to ableism, parents may spend time and energy either advocating and educating the community about their child’s disability or hiding and ignoring it (Neely-Barnes et al., 2010). In addition, *experiential avoidance*, defined as an unwillingness to remain engaged in unpleasant private events (feelings, thoughts, and bodily sensations) and feeling “trapped” in repeated attempts to control the contexts in which these experiences may arise by changing, predicting, or avoiding them (Fledderus et al., 2010; Ruiz, 2010). Experiential avoidance is associated both with stress and distress in the general population (Ruiz, 2010) and in parents of children with disabilities specifically (Sairanen et al., 2018). Parental stress and distress are known to influence children’s well-being, development, treatment outcomes, and behavioral problems (Barroso et al., 2018; Lessenberry & Rehfeldt, 2004). Furthermore, children’s behavioral problems and parental stress and distress have been found to influence each other in a mutual relationship (Neece et al., 2012). Swedish interest organizations for neurodevelopmental disorders have identified support for family members as one of the most important areas in need of future development (Hirvikoski, Jonsson, et al., 2015; Hirvikoski, Waaler, et al., 2015; Lappalainen et al., 2021). Likewise, the international literature has acknowledged an urgent need for the systematic development and evaluation of interventions aimed at improving the mental health of parents of children with disabilities (Dykens & Lambert, 2013; Lindo et al., 2016; Whittingham, 2014).

Acceptance and Commitment Therapy (ACT) is a contextual cognitive behavioral therapy. According to several meta-analyses, it is an effective treatment for a wide variety of psychological conditions, including depression, stress, and anxiety (A-Tjak et al., 2015; Ruiz, 2010). The main therapeutic goal is to increase *psychological flexibility* (PF), which in turn leads to a decrease in experiential avoidance (Hayes et al., 2009). As a functional behavior class, PF is a repertoire of behaviors characterized by the acceptance of inner experiences and flexibly attending to environmental stimuli with consideration to the actual context and to one’s personal values (Hayes et al., 2009). The PF model used in ACT has six interactive processes, as well as a psychopathological (counter-) expression. The curative processes are: (1) mindful attention to the present moment, (2) cognitive defusion (using helpful, workable ways of relating to thoughts and other private experiences), (3) experiential acceptance, (4) perspective-taking and deictic relational frames, (5) sense of direction in life through values, (6) commitment to value-based behaviors (Hayes et al., 2009). This therapeutic approach does not necessarily seek to eliminate symptoms: its indispensable goal is instead to increase individuals’ self-awareness and practical functioning (workability) (Villatte et al., 2015). The learned behaviors that lead to PF are seen as pivotal skills that determine the long-term effects of stress and distress as they promote resilience and adaptive coping (Leeming & Hayes, 2016). A high level of PF is associated with both greater well-being and better family functioning (Lappalainen et al., 2021; Prevedini et al., 2020). In addition, high parental PF has been linked to more adaptive and effective parenting practices, as well as higher-quality and more-frequent parent–child interactions (Leeming & Hayes, 2016; Shea & Coyne, 2011). ACT interventions for parents address mindful and flexible parenting, experiential acceptance of the child and oneself, and acknowledgment of the role of language in unhelpful parenting patterns. Behavioral changes are grounded in the ability to take an observer’s stance on one’s own and the child’s behavior, allotting time for an analysis of the situation, and then making the choice to use flexible, value-directed parenting actions or take self-care measures (Prevedini et al., 2020). The assumption is that automatic, punitive, rigid parenting practices will diminish, and skills such as engaging in flexible attention (e.g., perspective-taking, mindfulness, and use of reinforcement) will increase (Cheron et al., 2009; Prevedini et al., 2020). Parents’ increased PF may “launch a positive spiral” that, in turn, contributes to greater satisfaction in parenting and improved welfare for the entire family (Leeming & Hayes, 2016). Despite these positive results, only a few pilot studies and small-scale randomized controlled trials (RCT) have examined ACT interventions in the context of parenting autistic children (Blackledge & Hayes, 2006; Byrne et al., 2020; Fung et al., 2018; Gould et al., 2018; Hahs et al., 2019). The preliminary results are promising and point to, for example, improvements in PF, experiential avoidance, stress, anxiety, and depression. According to a recent systematic review (Juvin et al., 2021), ACT is a promising treatment for distress in parents of autistic children. However, the included studies have used relatively small sample sizes (3–33 participants). Another recent systematic review concluded that ACT appears promising for helping parents of children with different disabilities manage stress and distress (Byrne et al., 2020). Furthermore, internet-delivered ACT has been found to be effective in helping parents of children with chronic conditions and disabilities (Lappalainen et al., 2021; Sairanen et al., 2019); and likewise, an ACT-enriched parent training program has been reported to be superior to traditional parenting training for parents of children with CP or acquired brain injury (Brown et al., 2015; Whittingham et al., 2016). Given the common reference point of parenting a child with any type of disability, and for the sake of enhancing future upscaling and implementation, a review (Byrne et al., 2020) and a proof-of-concept paper (Prevedini et al., 2020) point to *transdiagnostic* ACT protocols as a possible alternative to diagnosis-specific manuals. This is logical, as ACT is a transdiagnostic therapy (Dindo et al., 2017). However, we lack transdiagnostic research and common ACT protocols for parents to children with disabilities despite the apparent benefits of such an approach (e.g., cost-effectiveness and logistics). Lastly, a treatment may be supported by many well-controlled studies (i.e., have good efficacy), but may still not be feasible if there are obstacles to successful implementation. The feasibility of an intervention (program completion, treatment credibility, etc.) predicts both treatment outcomes and successful implementation (Foster & Mash, 1999). The extent to which participants view the intervention as credible and useful in a given context, along with their beliefs on treatment success, are commonly used indicators of treatment outcome in treatment-focused research (Delsignore & Schnyder, 2007; Devilly & Borkovec, 2000). High treatment credibility and expectancy predict better attendance, treatment adherence, and motivation, as well as greater therapeutic gains (Bowen et al., 2009; Nock et al., 2007). Examining the feasibility from the perspectives of both the participant and the treatment provider sets the stage for successful implementation. Treatment providers who find the treatment credible adhere more consistently to the treatment manual; this, in turn, contributes to greater therapeutic advantages (Bowen et al., 2009). In addition, it is beneficial to examine feasibility in a clinical context prior to launching a costly RCT, which requires substantial resources.

This study serves to both examine the feasibility of ACT treatment for parents of children with different disabilities for successful implementation in a clinical context and to test whether the concept is ready for a RCT. The objective of the current study was to examine Navigator ACT, a novel, manualized, trans-diagnostic group treatment which was recently developed at Habilitation and Health, an outpatient habilitation service in Stockholm, Sweden. The goal of Navigator ACT is to increase the PF and psychological well-being of parents of children with disabilities. The primary aim of this study was to systematically evaluate feasibility (completion rate, factors predicting treatment completion, treatment credibility, expectancy, usefulness, and satisfaction) of Navigator ACT in the context of clinical outpatient habilitation services, as a preliminary step before conducting a RCT and possible large-scale implementation. The secondary aim was to evaluate preliminary treatment outcomes with respect to parental stress and distress (depression, anxiety, and experiential avoidance), mindfulness skills, as well as the index children's difficulties and strengths to see how parents perceive their child’s behavior before and after the treatment.

## Methods

### Study Design and Setting

The data for this open feasibility study were collected during 2016–2018 as part of clinical work at six habilitation centers (outpatient health care disability service clinics) in both Stockholm and Uppsala regions. The Regional Ethics Committee of Stockholm approved the study (2016/526-21-1, 2016/526-31/1). Participants signed a written informed consent before taking baseline measurements and allocating them to the treatment.

### Recruitment Process and Enrollment of Participants

We used the centers’ websites, course catalogs, and informational brochures to recruit 143 parents for a screening interview and needs assessment that lasted 20–30-min. Treatment participation was subject to the index child having contact with disability services, i.e., a diagnosed disability or a not yet diagnosed but identified severe developmental delay (for preschool children). The inclusion criteria were as follows: (1) parents to children aged 0–17 years with a disability or severe developmental delay, (2) symptoms of stress, depression, or anxiety associated with the child’s disability, (3) adequate knowledge of the Swedish language, and (4) the ability to participate in all sessions. Parents who exhibited severe psychiatric problems (e.g., suicidal ideation) were excluded and referred to specialized psychiatric services. If needed, the patient’s case files could be consulted. An information meeting was arranged for parents who matched the inclusion criteria. However, the final decision on inclusion took place only after the informed consent was signed and standardized self-rating questionnaires were administered, including the Hospital Anxiety and Depression Scale (HADS*)* to assess the level of distress. Due to the importance of the individual process, couples (i.e., parents of the same child) were advised to participate in different groups. Out of the interviewed applicants, 94 individuals met the inclusion criteria and were allocated to treatment. Because we were investigating feasibility from the treatment provider perspective as well, fifteen group leaders (n = 15) took part in the study. These were health care professionals who were enrolled in the Navigator ACT group leader training: psychologists (n = 6), social workers (n = 8), and one special education teacher. Training participants were selected based on the applicants’ professional credentials, previous experience, and motivation. Three of the authors of this paper developed and held the seven-day, 45-h group leader training that extended over 6 months and aimed at teaching Navigator ACT facilitator skills and increasing treatment fidelity. The training consisted of lectures, clinical supervision, and clinical practice (i.e., holding a Navigator ACT group). The manual’s developers provided supervision during training after each treatment session, which allowed them to monitor skill acquisition and collect user feedback for the purpose of reviewing and editing the manual. Clinical supervision was only provided during the training period. For more details, see Table 1 Group leader training of Navigator ACT.

**Table 1:** Contents of the group leader training of Navigator ACT

Group leader background	Learning activities	Content and extent
*Group leaders* Health care professionals in the disability (habilitation) services with a higher education degree (Magister/Master’s), e.g., licensed psychologists, social workers, speech- and language therapists or special education teachers *Group leader training educators and supervisors* Experienced clinical psychologists with several years of theoretical and clinical work with ACT	*Theory* Lectures in ACT; Navigator ACT model; stress/distress in parents of children with disabilities; practical issues (recruiting, needs assessment, group preparations); group leader role; mastering ACT in clinical conversations; review of each session *Experiential learning* Experiential exercises; metaphors; role-playing; parallel ACT-process through personal professional development *Supervision* Reflections, discussions and feedback regarding clinical training, in-depth practice of skills *Homework* Four-step mindfulness training, values as a group leader, work-related triggers, acceptance-and defusion. Completion of the same homework as parents *Required literature*: Harris (2013). The reality slaps. Finding peace and fulfilment when life hurts. Harris (2009). ACT made simple. Scientific articles	*Total training package* 7 days inclusive supervision reaching over 6 months. Parallel clinical practice *Lectures/experiential workshops*: 7 h introductory day followed by 5 × 3.5 h workshops covering the content of each session *before* facilitation of a group session. (Total: 24.5 h) *Clinical training*: Facilitation of the first group together with a colleague inclusive recruiting and needs assessment. (Total: 40 h) *Supervision*: In small groups *after* each group session. 5 occasions, each 2.5 h (Total 12,5 h)

### Navigator ACT Treatment

Navigator ACT is a transdiagnostic, manualized treatment developed by Habilitation and Health, Region Stockholm, in Stockholm, Sweden. It was originally modified from the manual ACT to Deal with Stress and Promote Health (Livheim, 2008) following ten years of experience with ACT groups for parents of autistic children as part of outpatient habilitation services in Stockholm. In addition, the treatment outline was inspired by Lisa Coyne’s (Harvard Medical School) protocol for ACT groups for parents of autistic children and other relevant literature. Two ACT-trained psychologists with extensive experience in facilitating ACT groups for parents of autistic children, together with the Navigator ACT project team, contributed to developing the final treatment protocol. The Navigator ACT manual is based on the general working processes of ACT, with its main therapeutic aim being to increase parental PF. The treatment consists of five sessions (3.5 h each) in a closed group and one booster session (2.5 h), resulting in a total of 20 h of treatment. Each of the five sessions has a theme, which is addressed through experiential exercises, metaphors, paradoxes, role-play, imaginary exposure, and psychoeducation. Homework is an essential part of the group work (See Table 2 for Session, theme, main topics and homework in Navigator ACT). The optimal group size is 8–16 parents. Two health care professionals facilitate the group, following the instructions and example dialogs from the structured treatment manual.

**Table 2:** Session, theme, main topics and homework in Navigator ACT

Session	Theme	Main topics	Homework
1	“Where am I?”	Parenting a child with disability; psychoeducation on parenting stress, grief and the importance of rest; creative hopelessness; workability of used coping strategies; control and avoidance; acceptance as an alternative	Guided mindfulness exercises: mindful observing of the child; noticing struggle and acceptance; action plan: recovery and rest
2	“What is important to me?”	Impact of language in suffering; healthy distance to inner experiences; mindfulness in everyday chores, play and activity; valued areas of life; values and goals; acceptance of the child with disability; self-compassion	Guided mindfulness exercises; mindfulness in everyday tasks, play and activity; noticing struggle and acceptance; Parent-Navigator: create a route; action plan: recovery and rest
3	“What can stop me?”	Values-work: “Navigator for parenting and life”; identification and management plan for inner and outer barriers to values-based actions; verbal rules as barriers, triggers in challenging parenting situations	Guided mindfulness exercises; mindfulness in everyday tasks, play and activity; action plan: recovery and rest; Parent-Navigator; identification of triggers
4	“What do I need to do?”	Observer-perspective and perspective change; “throw the anchor”- mindfulness and experiential acceptance in challenging parenting situations; doing what matters: committed action plan	Guided mindfulness exercises; mindfulness in everyday tasks, play and activity; action plan: recovery and rest; action plan: valued activities and managing barriers
5	“What do I promise myself?”	Create energy-balance: change or accept daily activities; commitment to values-based parental choices and actions; self-care: mindfulness in pleasant situations; self-compassion; psychologically flexible parenting	All homework from previous sessions; letter to myself; Navigator ACT for life!

## Measures

### Background and Demographic Variables

The background information and demographics were obtained from the modified version of the Current Life Situation Questionnaire (CLSQ) (Hirvikoski et al., 2009). Participants were asked to answer questions regarding their gender, educational level, occupational and relationship status, and parental role (full-time or part-time), as well as ongoing or previous experience with counseling and psychoactive medication. The CLSQ included questions regarding the index child: e.g., their main and comorbid diagnoses and the number of children (with and without disabilities) in the family. These measurements are categorized and presented in Tables 3 and 4.

**Table 3:** Demographic information of parents enrolled in the study divided into completers and non-completers

n = 94	*n* = total included (%)	*n* = out of 69 completers (%)	*n* = out of 25 non-completers (%)	Test statistics
Gender	85 (90.4) Female 9 (9.6) Male	62 (89.1) Female 7 (10.9) Male	23 (92.0) Female 2 (8.0) Male	*n.s*
Parent disability	84 (89.4) No disability 10 (10.6) Disability	44 (92.8) No disability 5 (7.2) Disability	20 (80.0) No disability 5 (20.0) Disability	*n.s. (p* = *0.07)*
University exam	54 (57.4) University exam 40 (42.6) No exam	44 (63.8) University exam 25 (36.2) No exam	10 (40.0) University exam 15 (60.0) No exam	*χ2* = 4.24, *p* < 0.05, V = 0.39
Occupational status	72 (76.6) Employed or student 22 (23.4) Unemployed or sick leave	51 (73.9) Employed or student 18 (26.1) Unemployed or sick leave	21 (84.0) Employed or student 4 (16.0) Unemployed or sick leave	*n.s*
Living with another adult	76 (80.9) Yes partnership 18 (19.1) Single parent	57 (82.6) Yes partnership 12 (17.4) Single parent	19 (76.0) Yes partnership 6 (24.0) Single parent	*n.s*
Caring for the child	82 (87.2) Full-time care 12 (12.8) Part-time or less	60 (87.0) Full-time care 9 (13.0) Part-time or less	22 (88.0) Full time care 3 (12.0) Part-time or less	*n.s*
Ongoing other counselling	66 (70.2) No counselling 28 (29.8) On-going counselling	43 (62.3) No counselling 26 (37.7) On-going counselling	23 (92.0) No counselling 2 (8.0) On-going counselling	*χ2* = 7.73, *p* < 0.05 *V* = 0.29
Previous counselling^a^	57 (64.0) Previous treatment 32 (36.0) No	41 (64.1) Previous treatment 23 (35.9) No	16 (64.0) Previous treatment 9 (36.0) No	*n.s*
Ongoing psychoactive medication	66 (69.9) No psychoactive medication 28 (30.1) On medication	51 (73.9) No psychoactive medication 18 (26.1) On medication	15 (60.0) No psychoactive medication 10 (40.0) On medication	*n.s*
Physical health	54 (57.4) Physical ill-health 40 (42.6) No physical health issues	38 (53.7) Physical ill-health 31 (46.3) No physical health issues	16 (64.0) Physical ill-health 9 (36.0) No physical health issues	*n.s*

^a^Missing n = 5

**Table 4:** Demographic information of the index children with disability divided into the children of completers and non-completers

n = 94	n = total included (%)	n = out of 69 completers (%)	n = out of 25 non-completers (%)	Test statistics
Gender^a^	66 (72.5) Boy 25 (27.5) Girl	38 (57.6) Boy 28 (42.4) Girl	18 (72.0) Boy 7 (28.0) Girl	*n.s*
Main disability	66 (70.2) ASD 28 (29.8) Other	50 (72.5) ASD 19 (27.5) Other	16 (64.0) ASD 9 (36.0) Other	*n.s*
Comorbid disability	55 (58.5) No other diagnosis 39 (42.5) Comorbid diagnosis	39 (56.5) No other diagnosis 30 (43.5) Comorbid diagnosis	16 (64.0) No other diagnosis 9 (36.0) Comorbid diagnosis	*n.s*
Number of children in the family	65 (69.9) Two children or less 29 (30.1) Three or more	50 (72.5) Two children or less 19 (27.5) Three or more	15 (60.0) Two children or less 10 (40.0) Three or more	*n.s*
Number of children with disability in the family^b^	74 (79.6) One with disability 19 (20.4) Two or more with disability	54 (79.4) One with disability 14 (20.6) Two or more with disability	20 (80.0) One with disability 5 (20.0) Two or more with disability	*n.s*
Number of children under 18 years	68 (72.3) Two children or less under 18 years 26 (27.7) Three or more under 18 years	52 (75.4) Two children or less under 18 years 17 (24.6) Three or more under 18 years	16 (64.0) Two children or less under 18 years 9 (36.0) Three or more under 18 years	*n.s*

^a^Missing *n* = 3, ^b^missing *n* = 1 (ASD = Autism Spectrum Disorder; Other = e.g., Celebral Palsy, Acquired Brain Injury, Multiple Disabilities; Intellectual Disability; Attention Deficit Hyperactivity Disorder; Physical Disabilities)

### Feasibility Measures

In this study, the primary assessments concerned the feasibility of Navigator ACT. The main assessment measures were treatment completion and satisfaction. The goal for good feasibility regarding completion was set a bit higher than previous clinical experience would point to, with the ambitious aim of having 75% of the participants complete the program (i.e., attending at least four out of five sessions and completing the post-measurement assessment at the end of the treatment (time point 2, T2). Treatment credibility and expectancy were measured with the Credibility and Expectancy Questionnaire (CEQ) (Devilly & Borkovec, 2000) before the start of treatment (at time point 1, T1) and at T2. C*redibility* refers to “face validity”: i.e., how believable, and logical the treatment appears. *Expectancy* refers to the patient’s belief in a positive treatment outcome. The CEQ contains two parts and six items, rated on a 1–9 Likert scale or a 0–100% scale, depending upon the item. The items in Part I are as follows: (1) How much sense does the treatment offered to you seem to make? 2) How sure are you that this treatment is going to reduce your distress? (3) How willing are you to recommend this treatment to a friend? 4) How much better are you going to feel at the end of the treatment? Part II repeats items 2 and 4, as the patient is asked to take a moment and then answer how they *intuitively* feel about the treatment. Higher CEQ scores reflect higher credibility and outcome expectancy (Devilly & Borkovec, 2000). *Session Evaluation Forms* (SEF) (Bramham et al., 2009; Hirvikoski, Jonsson, et al., 2015; Hirvikoski, Waaler, et al., 2015) were used to measure immediate satisfaction after each session. The SEF is composed of eight questions scored 1–9 on a Likert scale, aiming to measure the usefulness of the session content (questions 1–3), skills acquisition and readiness to use the skills (questions 4–6), and the benefits of sharing experiences with other parents (questions 7–8). In addition, a modified version of the Patient Evaluation Form (PEF) (Hesslinger et al., 2002; Hirvikoski et al., 2011) was used to evaluate the entire intervention at T2. The PEF consists of nine questions on a four-point Likert scale that targets whether the treatment had a clear focus and promoted ACT skills acquisition and future participation in similar groups. In addition, participants were provided with three open-ended questions that asked about how the treatment could be improved and what the parent could have done better and solicited open feedback. Finally, the treatment was evaluated by grading it on a four-point Likert scale from 0 to 3: fail, pass, well done, and very well done.

### Adverse Events and Serious Adverse Events

Group leaders recorded adverse events and serious adverse events were recorded in case report forms. An adverse event was defined as any kind of patient-reported difficulty or negative event. Serious adverse events were those requiring hospitalization. A possible association between the events and the treatment was evaluated after the end of data collection.

### Preliminary Outcome Measures

The effectiveness measures were included for a preliminary estimation of the treatment’s effects in order to assess whether further efficacy testing was recommended. Standardized self-rating questionnaires (Berry & Jones, 1995; Brown & Ryan, 2003; Cheron et al., 2009; Goodman, 2001; Zigmond & Snaith, 1983) with good psychometric properties were administered at T1 (0–2 weeks before treatment), at T2 (0–2 weeks after the treatment), and at time point 3, T3 (3–4-month follow-up). Four measures were used. (1) The Parental Acceptance and Action Questionnaire (PAAQ; Cheron et al., 2009) is a 15-item scale that targets experiential avoidance in the parenting context. The PAAQ is divided into subscales on *Unwillingness* (parental avoidance of the child’s emotional experiences) and *Inaction* (parental avoidance of action-taking in the context of the child’s negative emotional experiences). The inaction scale has nine items and the unwillingness scale six items, each scored from 1 to 7. Higher scores indicate higher experiential avoidance, and lower scores indicate greater PF (Cheron et al., 2009). The scale was translated into Swedish using a translation/back-translation procedure. Cronbach’s alpha for the PAAQ in this study was α = 0.66. (2) The Mindfulness Awareness Attention Scale (MAAS; Brown & Ryan, 2003) is a 15-item scale (scored from 1 to 6) where higher scores reflect greater mindfulness, and lower scores indicate psychological distress. In this study, MAAS showed an internal consistency of α = 0.87 (3) The Parental Stress Scale (PSS) (Berry & Jones, 1995) is an 18-item measure of parental stress scored from 1 to 5, with sum scores ranging from 18 to 90. The scale is composed of four subscales: rewards, stressors, lack of control, and dissatisfaction in parenting. Higher scores imply greater parental stress. The internal consistency for the PSS in this study was α = 0.77. (4) The Hospital Anxiety and Depression Scale (HADS) (Zigmond & Snaith, 1983) is a 14-item rating scale to measure clinical depression and anxiety consisting of the seven-item HADS-A (anxiety) and seven-item HADS-D (depression) subscales, with each item scored from 0 to 3. A subscale score ≥ 8 represents clinical anxiety *or* depression, and a score ≥ 14 for the combined scale indicates that both disorders are present (Brennan et al., 2010). In this study, Cronbach’s alpha for HADS was 0.85. (5) The Strengths and Difficulties Questionnaire (P4-17 SDQ) (Goodman, 2001) is a 25-item, caregiver-administered scale to assess difficulties and strengths in children aged 4–17 years. Four of the subscales reflect difficulties (emotional, conduct, peer, and hyperactivity/inattention), and one reflects a strength (prosocial behavior). Higher scores reflect both greater difficulties and strengths. In this study, the internal consistency of the SDQ was α = 0.65.

### Treatment Provider Measures

The group leaders filled out a slightly adjusted version of the CEQ (Devilly & Borkovec, 2000) before they held their first group session (at T1) and again after completing the last day of their training (at T2). The questions concerned: (1) Navigator ACT’s credibility and outcome expectancy as a treatment for parents of children with disabilities (Part I), and the credibility of the Navigator ACT group leader training as an ACT training program, and the expectancy of gains in personal competency (Part II). Part I contained four items and Part II five items, all rated 1–10 on a Likert scale. In addition, satisfaction with and perceived usefulness of the group leader training was assessed using a modified version of the session evaluation form (SEF) at the end of the theoretical portion of the training (sessions 2–6) and before each supervision session. The questions regarded learning theory and practical (Navigator) ACT skills, the usefulness of the current learning session, and the benefits of being able to share experiences with other group leaders.

### Statistical Analysis

Statistical analyses were performed using IBM SPSS Statistics version 26. Data were screened and described using descriptive statistics. There were a small number of missing data points (n = 4). Treatment completers were compared to non-completers using Chi-square, along with Cramer’s V for effect size (Cohen, 1988). The effect sizes were interpreted as follows: weak (0.10–0.20), moderate (0.20–0.40), relatively strong (0.40–0.60), strong (0.60–0.80), or very strong (> 0.80) (Cohen, 1988). The CEQ had items using both a Likert scale and percentages; therefore, the items in percentages were transformed to match the items on the 0–10 Likert scale. The values of 40%, 50%, and 60% were all assigned a score of 5 (Nock et al., 2007). The PEF had open-ended questions, which were analyzed using an inductive thematic data analysis by the first author. The reoccurring phrases were grouped together and coded into themes. The emerged themes were then validated by another researcher (PL) and finally accepted after a consensus discussion in the research group. The effectiveness-related data was analyzed from T1 to T2 and from T1 to T3 with separate series of repeated-measures ANOVA (rmANOVAs). Outliers in the continuous variables were analyzed using boxplots and were generally few. The rmANOVAs were conducted *per protocol*: i.e., only participants that attended at least four of the five treatment sessions and completed the post-measurement assessments were included. Mauchly’s test of sphericity was also conducted. In the few cases of violation of sphericity, Greenhouse–Geisser was used (Tabachnick & Fidell, 2019) The effect sizes were expressed as partial eta squared (*η*_*p*_^*2*^) and interpreted as 0.01–0.05 = small, 0.06–0.13 = medium, and 0.14 and up = large (Cohen, 1988). All the statistical analyses were planned a priori*,* and the alpha level was set at 05.

## Results

### Background and Demographic Variables

**Fig. 1:**
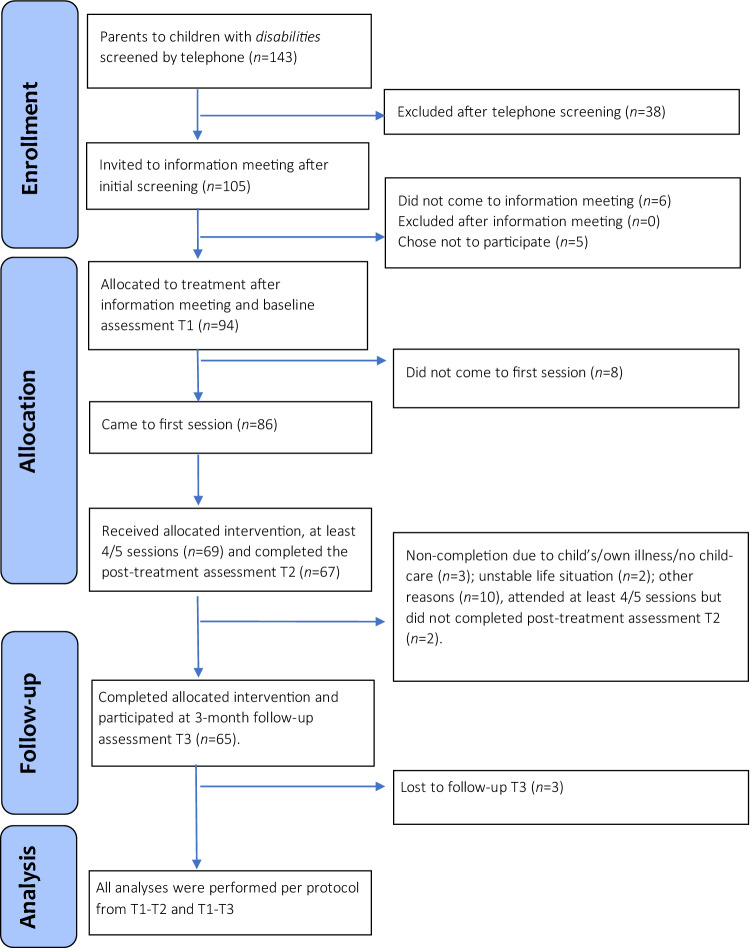
Flowchart of parents enrolled in the study. Microsoft Word, 2016

Out of the 143 screened parents, 94 (85 mothers and 9 fathers) were allocated to the treatment. Eight of the allocated parents never came to the first session, resulting in 86 parents starting the treatment. The details regarding enrollment, allocation, and completion count are shown in Fig. 1 Flowchart of parents enrolled in the study.

The participating mothers and fathers ranged in age from 27 to 55 (M = 41.4, SD = 6.18), parenting at least one child aged 2–17 years (M = 8.26, SD = 4.08) with a disability (including ASD, ID, ADHD, and motor disabilities). At T1, the participating parents exhibited mild clinical depression on average (HADS-D subscale M = 9.30, SD = 3.41), moderate to severe anxiety (HADS-A subscale M = 13.3, SD = 4.13) and parenting stress (PSS M = 48.3, SD = 10.72;). Demographic information, divided into completers and non-completers, is presented in Table 3 for parents and in Table 4 for the index children.

### Feasibility

**Fig. 2:**
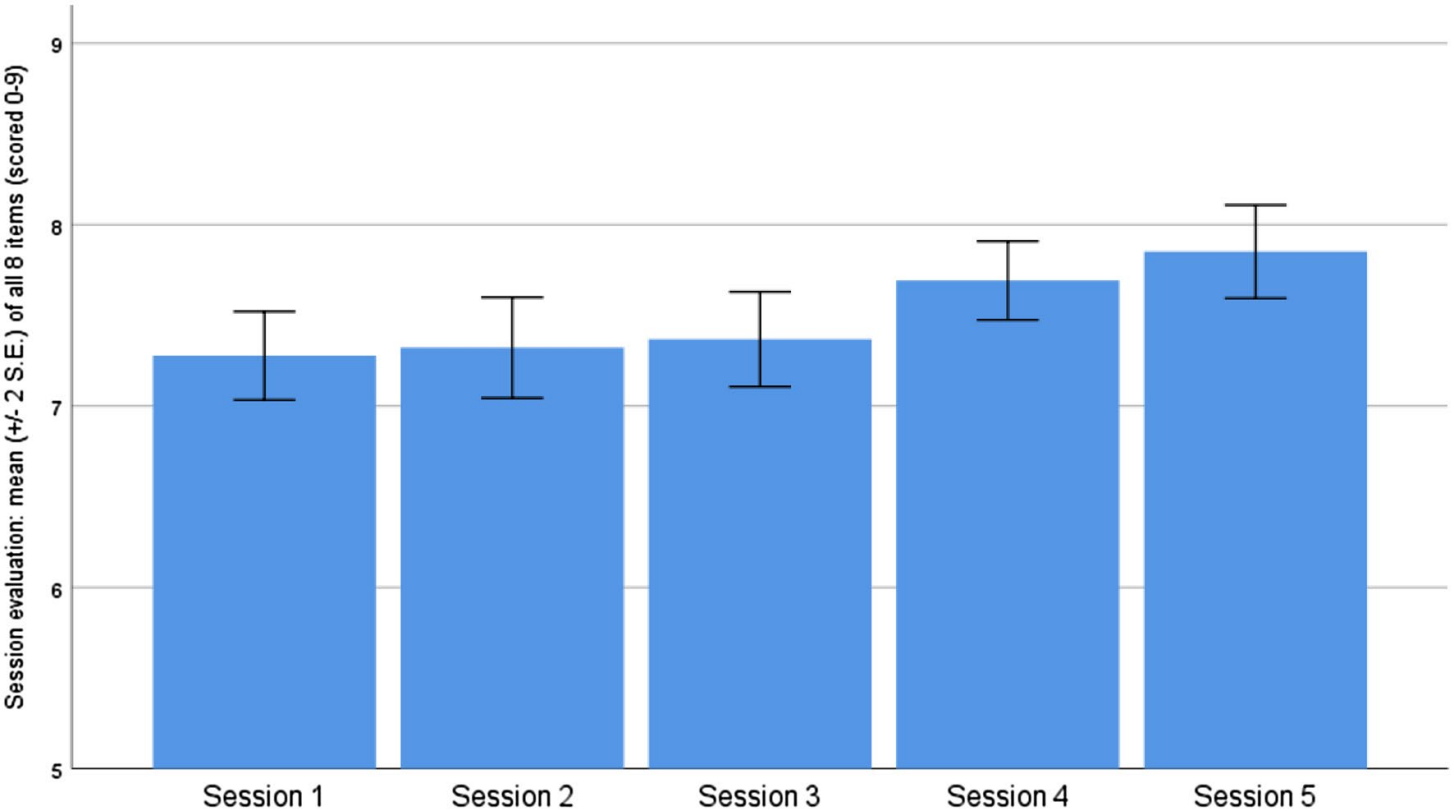
Mean score of treatment satisfaction through session evaluations, SEF (scored1–9 for each session). IBM SPSS Statistics, version 26

Of the 86 parents that came to the first session, 69 (80%) attended at least four out of the five sessions (two parents who completed at least four sessions did not complete the post-assessment). There were no significant differences between completers (n = 69) and non-completers (n = 25) in terms of demographic data at baseline, with the exception of two variables. Completers were more likely to have university education (*p* < 0.05) and to be participating in ongoing counseling (*p* < 0.05) compared to non-completers. The most common reasons for non-attendance were unpredictable and sudden stressful events, childcare problems, too many appointments with disability services, or illness (child’s or parent’s). Complete dropout occurred mainly due to childcare problems, illness, or another type of crisis. Regarding the credibility and expectancy of the intervention, parents reported an average of M = 6.7 (SD = 1.32) out of 9.0 at T1, and M = 7.3 (SD = 1.19) at T2 on the CEQ for the average of all items. Session satisfaction (SEF, average of all items) ranged from M = 7.20 (SD = 0.89) for the first session to M = 7.84 (SD = 0.84) for the last session, on a scale of 1 to 9. Figure 2 Mean scores of treatment satisfaction through session evaluations (SEF). Further analysis of the SEF revealed that satisfaction with *session content* (items 1–3) ranged from M = 6.81 (SD = 1.24) to M = 7.80 (SD = 1.08), satisfaction with *skills acquisition* (items 4–6) ranged from M = 7.09 (SD = 1.02) to 7.60 (SD = 1.04), and satisfaction with *sharing experiences with other parents* (items 7–8) ranged from M = 7.30 (SD = 1.36) to 8.22 (SD = 0.89). With respect to the evaluation of the treatment as a whole at T2 (PEF), the results showed high satisfaction both with the treatment and with the learning of ACT skills. Scores on the PEF ranged from the highest mean score *M* = 3.97 (*SD* = 0.02) out of a maximum of 4 for the item “the treatment had a clear focus on parents to children with disabilities” to the lowest score of M = 2.97 (SD = 0.13) for the ACT-skills related item “I do things that are important to me more often” (engagement in values-based activities). In addition, participants gave a high grade of M = 2.6 on a scale of 0–3, indicating a strong assessment of the treatment being “well done.” The open-ended questions elicited responses from 47 parents regarding their suggestions for improvement and general feedback. The following themes emerged: 1) The treatment should increase the amount of time spent sharing with other parents (14 responses), 2) The participant should engage more in homework (16 responses), 3) Appreciation for group leaders and treatment content (16 responses). See Table 5 for the results of the thematic analysis of open questions.

**Table 5:** Results of the thematic analysis of open questions (Patient Evaluation Form, PEF)

Question 1 How the treatment could be improved?	Question 2 What I as a participant could have done better?	Question 3 General feedback
*Theme: More time for sharing/talking with other parents*	*Theme: Should have done more homework*	*Theme: Appreciation for group leaders and group content*
Examples: “Wish to have more discussions in a group to share similarities and differences in our children.” “A few more sessions and longer time to discuss with each other and share experiences. It took 3 sessions until we really started to talk with each other.” “More time for group discussions.”	Examples: “I should have been more meticulous and done more exercises.” “Should have done more homework, shared more.” “Should have made sure that I did more of the exercises in between the sessions.”	Examples: “Finally, a treatment for me and my well-being. It’s been a really good course! “A great group leaders and group. Great with concrete exercises/role-plays in combination with facts and mindfulness. Highest grade!” ” Totally awesome treatment. Give this to all parents!” “I want to continue to use these strategies that help me as a parent to gain increased well-being. It’s good even for my kids!”

### Adverse Events and Serious Adverse Events

Two serious adverse events were reported (e.g., vehicle accident) but were judged to be unrelated to the treatment per se.

### Preliminary Outcome Measures

The rmANOVAs showed a significant reduction in experiential avoidance (PAAQ) from T1 to T2 (*p* < 0.001) with large effect size η_*p*_^*2*^ = 0.19. When the PAAQ subscales were analyzed separately, a larger change was observed in the Inaction subscale *(p* < 0.001, *η*_*p*_^*2*^ = 0.21) than in the Unwillingness subscale (*p* < 0.05, *η*_*p*_^*2*^ = 0.08). Furthermore, improvements were shown in mindfulness skills (MAAS, *p* < 0.001), depression (HADS-D, *p* < 0.001), and anxiety (HADS-A, *p* < 0.001) with large effect sizes. At T3, the effects remained stable. See Table 6 for the parent-related descriptive statistics and results of the rmANOVAs, and Table 7 for the child-related variables and rmANOVAs. The decline in parental stress (PSS) was not statistically significant at T2 at the total scale level but showed a significant change at T3 (*p* < 0.05). However, one of the subscales of the PSS—satisfaction in parenting—was significant at both T2 (*p* < 0.05) and T3*, (p* < 0.001), indicating increased parental satisfaction.

**Table 6:** Means and standard deviations (SD) for parent-related outcome variables at T1–T2 and T1-T2-T3, repeated measures ANOVA (rmANOVA), p-values and effect sizes (η_p_^2^)

*n* = 94	Baseline T1 *n* = 69 M (SD)	Post T2 *n* = 67 M (SD)	Follow-up T3 *n* = 64 M (SD)	rmANOVA T1-T2 M (SD)	rmANOVA T1-T2-T3 M (SD)
PAAQ -15 total EA	65.92 (11.8)	60.31 (10.7)	58.25 (11.6)	*F*_(1,64)_ = 14.55 *p* < *0.001* η_*p*_^*2*^ = 0.19	*F *_(2,120)_ = 12.82 *p* < *0.001* η_*p*_^*2*^ = 0.18
PAAQ EA-unwillingness	29.89 (5.96)	28.38 (5.66)	27.49 (6.67)	*F *_(1,64)_ = 5.16 *p* < *0.05* η_*p*_^*2*^ = 0.08	*F *_*(*1.8, 120)_ = 4.94 *p* < *0.05* η_*p*_^*2*^ = 0.08
PAAQ EA-inaction	36.03 (8.06)	31.92 (6.64)	30.75 (7.60)	*F *_*(*1, 64)_=16.67 *p* < *0.001* η_*p*_^*2*^ = 0.21	*F *_(2,120)_ = 14.03 *p* < *0.001* η_*p*_^*2*^ = 0.19
MAAS mindfulness	3.01 (0.67)	3.45 (0.76)	3.64 (0.73)	*F*_(1,66)_ = 20.87 *p* < *0.001* η_*p*_^*2*^ = 0.24	*F *_*(*2, 124*)*_ = 24.89 *p* < *0.001* η_*p*_^*2*^ = 0.29
HADS total	22.57 (6.47)	16.46 (6.18)	15.71 (7.21)	*F *_(1,66)_ = 60.44 *p* < *0.001* η_*p*_^*2*^ = 0.48	*F*_(2,124)_ = 40.85 *p* < *0.001* η_*p*_^*2*^ = 0.40
HADS depression	9.30 (3.41)	6.51 (3.38)	5.97 (3.44)	*F*_(1,66)_ = 43.66 *p* <* 0.001* η_*p*_^*2*^ = 0.40	*F *_(2, 124)_ = 33.64 *p* <* 0.001* η_*p*_^*2*^ = 0.35
HADS anxiety	13.27 (4.13)	9.96 (3.81)	9.75 (4.37)	*F *_(1,66)_ = 50.87 *p* < *0.001* η_*p*_^*2*^ = 0.44	*F*_(2,124)_ = 32.38 *p* < *0.001* η_*p*_^*2*^ = 0.34
PSS total	48.25 (10.72)	46.54 (9.10)	45.32 (10.9)	*n.s*	*F*_(1.8, 124)_ = 3.11 *p* <* 0.05* η_*p*_^*2*^ = 0.05
PSS rewards	11.06 (4.29)	11.00 (3.87)	10.71 (4.18)	*n.s*	*n.s*
PSS stressors	20.82 (4.76)	20.04 (4.49)	19.83 (4.95)	*n.s*	*n.s*
PSS lack of control	7.77 (2.79)	7.09 (2.60)	7.18 (2.68)	*n.s*	*n.s*
PSS dissatisfaction in parenting	8.16 (2.62)	7.28 (2.68)	6.83 (2.75)	*F*_(1, 63)_ = 6.76 *p* < *0.05* η_*p*_^*2*^ = 0.10	*F*_(1.81, 118)_ = 10.46 *p* < *0.001* η_*p*_^*2*^ = 0.15

*PAAQ-15* Parental Acceptance and Action Questionnaire, 15-item version; *MAAS* Mindfulness Awareness and Attention Scale; *HADS* Hospital Anxiety and Depression Scale; *PSS* Parental Stress Scale

*p*-values in italics denote statistically significant changes in outcomes

**Table 7:** Means and standard deviations (SD) for child-related outcome variables at T1–T2 and T1-T2-T3, repeated measures ANOVA (rmANOVA), p-values and effect sizes (η_p_^2^)

*n* = 94	Baseline T1 *n* = 69 M (SD)	Post T2 *n* = 67 M (SD)	Follow-up T3 *n* = 62 M (SD)	rmANOVA T1-T2 M (SD)	rmANOVA T1-T2-T3 M (SD)
SDQ- total difficulties	19.51 (6.94)	17.71 (6.36)	17.35 (6.96)	*F*_(1, 64)_ = 10.63 *p* < *0.05* η_*p*_^*2*^ = 0.14	*F*_(2,118)_= 8.73 *p* < *0.001* η_*p*_^*2*^ = 0.13
SDQ STRENGHT Pro-social behavior	4.91 (2.51)	5.58 (2.67)	5.77 (2.47)	*F *_(1, 64)_ = 11.07 *p* < *0.001* η_*p*_^*2*^ = 0.15	*F*_(2, 118)_ = 7.34 *p* < *0.001* η_*p*_^*2*^ = 0.11
SDQ Emotional problems	4.34 (2.65)	3.65 (2.53)	3.90 (2.62)	*F*_(1,64)_ = 9.50 *p* < *0.05* η_*p*_^*2*^ = 0.13	*F*_(1.8, 118*)*_= 4.70 *p* < *0.05* η_*p*_^*2*^ = 0.07
SDQ Conduct problems	3.85 (2.40)	3.38 (2.19)	2.95 (2.29)	*F*_(1,64)_ = 6.51 *p* < *0.05* η_*p*_^*2*^ = 0.09	*F *_(1.81, 118)_=12.80 *p* = *0.001* η_*p*_^*2*^ = 0.18
SDQ Inattention/hyperactivity	6.75 (2.66)	6.34 (2.73)	6.17 (2.74)	*n.s*	*n.s*
SDQ Peer problems	4.57 (2.23)	4.34 (1.91)	4.33 (2.06)	*n.s*	*n.s*
SDQ Impact score	5.28 (2.71)	4.60 (2.76)	4.48 (2.94)	*F *_(1, 64)_=5.60 *p* < *0.05* η_*p*_^*2*^ = 0.08	*F *_(1.7, 118)_ = 4.11 *p* < *0.05* η_*p*_^*2*^ = 0.07
SDQ Total burden score	1.52 (0.62)	1.31 (0.68)	1.15 (0.81)	*F *_(1, 64)_ = 6.66 *p* < *0.05* η_*p*_^*2*^ = 0.09	*F*_(1.7,118)_ = 12.46 *p* < *0.05* η_*p*_^*2*^ = 0.17

*SDQ* Strenghts and Difficulties Questionnaire

Regarding the index child’s difficulties and strengths (SDQ), there were significant reductions in the child’s total difficulties (*p* < 0.05), the impact of the child’s difficulties on the family (*p* < 0.05), and the experienced burden of care (*p* < 0.05). When the subscales were analyzed separately, a significant reduction was seen in the child’s emotional problems (*p* < 0.05) and conduct problems (*p* < 0.05), coupled with an increase in prosocial behaviors (*p* < 0.001). All child-related measures were maintained or improved at T3.

### Treatment provider measures

Fifteen treatment providers completed the adjusted CEQ, scored 1–10. The overall credibility and expectancy were M = 9.07 (SD = 0.37) for the Navigator ACT treatment (part I), and M = 9.59 (SD = 0.50) for the group leader training (part II). In addition, the treatment providers satisfaction with the course sessions ranged from M = 7.00 (SD = 0.74) to M = 7.49 (SD = 0.93) out of maximum 10 (SEF).

## Discussion

The main aim of this study was to determine the feasibility of the novel, transdiagnostic manualized Navigator ACT group intervention, in the context of publicly funded outpatient habilitation services as measured using treatment completion and user satisfaction. In addition, the preliminary outcome of the Navigator ACT treatment was evaluated using measures of parental well-being and child outcomes, such as the emotional and behavioral problems of the child.

### Feasibility

The goal of “good feasibility” (at least 75% of participants attending four out of five sessions) was met. In a disability services context, the 80% of participants who completed the program can be considered very good, as last-minute cancellations and treatment dropouts are common among parents of children with disabilities (Ballantyne et al., 2019), as well as in health care treatment in general (Fenger et al., 2011). Our completion requirement (attending at least four out of five sessions) was quite strict, meaning that parents who missed more than one session were considered non-completers even if they attended most of the treatment sessions and completed the assessments at T1, T2, and T3. Individual psychotherapeutic treatments (including ACT) usually have a dropout rate of between 15 and 30% (Ong et al., 2018). In earlier studies, demographic variables such as living alone, low educational attainment, being unemployed, psychiatric comorbidities, and current treatment with psychotropic medication have been associated with both missed treatment sessions and dropout (Fenger et al., 2011); being a female and caring for a child have also been linked to missed sessions. but not to dropout (Fenger et al., 2011). The participants in our sample reported “practical reasons,” for missed sessions and dropping out, such as childcare problems, illness, and sudden stressful events. When we analyzed and compared the demographic variables and characteristics of completers and non-completers, we generally did not find significant differences. As in the above-mentioned previous study (Fenger et al., 2011), the completers in our study had a higher educational attainment (university exam) than non-completers. Furthermore, the completers more often had other ongoing support or counseling during the treatment. The nature of “other counseling” was not specified in the questionnaire. According to the feedback from the group leaders, “other support” could have been anything from a supportive discussion with a social worker to coaching or psychological counselling. However, 70% of participants were not receiving other counseling during the time they participated in the Navigator ACT treatment. In addition, the Chi-square test approached significance for parental disability, (p = 0.07).

We might speculate that our sample size (n = 94) was underpowered for making an analysis of completers vs. non-completers and thus risked reflecting type-II errors. For example, parents with their own disability may be at higher risk for non-completion and may possibly need additional support to complete the treatment.

Although there were few significant differences between completers and non-completers, the study participants were not problem-free. According to the sample’s characteristics, the typical participant was a stressed, middle-aged mother parenting an autistic child. She was mildly clinically depressed, moderately to severely anxious, and had chronic pain. The total mean score for depression and anxiety (M = 22.57, SD = 6.47, HADS) at T1 was well above the clinical cutoff (11 points) and above the average for the Swedish community sample (M = 8.53, SD = 6.54, HADS) (Lisspers et al., 1997). These symptomatic characteristics are similar to those that have been previously reported regarding stress, distress, and physical health issues in parents of children with disability (Dykens & Lambert, 2013; Hatton & Emerson, 2003; Neece & Chan, 2017; Theule et al., 2013). The characteristics of the sample (e.g., presence of clinical depression and anxiety) speak to the effectiveness of the structured screening process: i.e., it was motivated parents with clinical symptoms in the need of the intervention who received the treatment. Therefore, we recommend structured screening through a needs-assessment interview and a pre-intervention informational meeting as a regular part of the routine prior to the start of Navigator ACT treatment. However, what the sample characteristics also tell us is that we need to continue to develop the recruitment process and our informational brochures in order to better reach out to distressed fathers, parents with lower educational attainment, and parents of children with disabilities other than ASD.

Participants in this study reported good credibility and expectancy (CEQ) for the treatment at T1 (M = 6.7 out of 9.0, SD = 1.32), and thus Navigator ACT was considered logical, and parents expected a reduction in their symptoms. This may partly explain the participants’ good completion percentage and treatment adherence. According to several studies, high outcome expectations predict adherence to treatment, decrease the risk of dropout, and have an impact on treatment outcomes (Devilly & Borkovec, 2000; Nock et al., 2007; Thompson-Hollands et al., 2014). Furthermore, participants appreciated the treatment. Parents reported high satisfaction with all five sessions, with slightly increasing satisfaction in each session as the program went on. In addition, participants were satisfied with the treatment as a whole. They found the treatment to have a clear focus, to be meaningful and useful in the context of distress associated with parenting, and to be effective in teaching ACT skills. A clear majority of parents stated they would attend a similar treatment again in the future (M = 3.88 out of 4). One of the ACT skills–related items that the participants were least satisfied with (close to M = 3.0 out of 4) was experiential acceptance. Even though the mean still reflects relatively high agreement with the statement, it should be taken into consideration that *experiential acceptance* may need more attention during the treatment, as it is a difficult skill to master. The group leader feedback confirms the difficulties with this item. Moreover, according to our thematic analysis of open questions, the treatment protocol would be improved by allowing more time for sharing with other parents and placing greater emphasis on motivating participants to complete their homework. Other treatment studies emphasize the importance of homework for both treatment adherence and positive outcomes (LeBeau et al., 2013). In addition, when evaluating a new intervention, “first of all, do no harm” (Lilienfeld, 2007). Therefore, it is important to note that there were no treatment-related adverse effects or serious adverse effects reported.

### Treatment Providers

For the sake of investigating treatment feasibility and future large-scale implementation, we wanted to examine group leaders’ expectations and opinions regarding the treatment and their own group leader training. They found the treatment to be highly credible for the target group and they had high expectancy of parents completing the treatment and improving their mental health. The extensive group leader training may have increased the credibility of the treatment in the therapist’s eyes and affected their adherence to the manual; this, in turn, may have promoted positive outcomes in parents. According to previous research, treatment providers who find a treatment to be credible adhere more consistently to the manual and promote greater therapeutic advantages in patients (Bowen et al., 2009). In this study, the participating health care workers in the disability field were selected for ACT training after “passing” an interview that examined their credentials and motivation and receiving comprehensive information on the treatment and the research project—all of this may have promoted adherence to the manual. The group leaders appreciated the group leader training, finding it useful and effective in increasing their competency as ACT therapists. They had a good understanding of the information that was provided and were satisfied with their readiness to use the ACT strategies. However, they wished for more opportunities to share experiences with each other during the training. This may depend on the session evaluations being delivered directly after the “hectic” theoretical part of the training and not after the clinical supervision, where the leaders did have opportunities for sharing.

### Preliminary Outcome Measures

We evaluated the preliminary outcome of the Navigator ACT treatment, which seeks to increase PF and the psychological well-being of parents of children with disabilities, along with flexible and mindful parenting practices. Our preliminary results are in line with previous studies that have shown positive mental health changes in parents after an ACT intervention (Blackledge & Hayes, 2006; Byrne et al., 2020; Fung et al., 2018; Gould et al., 2018; Hahs et al., 2019). However, the majority of previous studies have focused on treatment groups for parents of children with the same diagnosis, mainly ASD. The current study lifts *transdiagnostic* ACT parent treatment groups as an option and confirms the results of earlier studies involving heterogeneous groups of parents of children with chronic conditions (e.g., Lappalainen et al., 2021; Sairanen et al., 2019). Over the past 40 years ACT has been successfully applied in a wide variety of settings for several different conditions, which makes it ideal in the context of transdiagnostic disabilities (Dindo et al., 2017).

We were able to identify significant parental improvements with regard to mindfulness (MAAS), parental experiential avoidance (PAAQ), depression (HADS-D), and anxiety (HADS-A), with large effect sizes. These findings are in line with those of several other studies that have looked at the use of ACT in supporting parents (Byrne et al., 2020; Juvin et al., 2021). The improvements we identified in our study were maintained at T3, a finding also consistent with previous research regarding the sustainable effects of ACT treatment (Kohtala et al., 2017). Surprisingly, the decline in overall parental stress (PSS) was not significant until T3. The subscale analysis showed that even then, the change was mainly due to an increase in one of the four subscales—satisfaction in parenting, which was already significant at T2.

Previous studies have proposed a transactional effect between parental involvement in ACT and an increase in children's psychological well-being and adjustment. Our results support this finding, as the index children’s difficulties—especially behavioral and emotional problems (SDQ)—declined significantly after their parents participated in the Navigator ACT treatment, while their prosocial behaviors had increased. At the baseline, these children with disabilities (e.g., ASD, ADHD, ID) showed a high level of additional difficulties, especially emotional and behavioral problems, which is in line with previous research concerning comorbid emotional and behavioral problems in children with disabilities (Emerson et al., 2010; Yorke et al., 2018). In this study, the most significant reductions in the index children’s difficulties (SDQ) concerned emotional and behavioral problems. Parental treatment targeting stress and distress has previously been associated with a decrease in behavioral problems in children due to the transactional relationship between parents’ stress/distress and children’s behavioral problems (Leeming & Hayes, 2016; Neece et al., 2012). Parents’ learning pivotal ACT skills such as experiential acceptance, flexible and mindful attentiveness to stimuli, verbal distancing (e.g., from negative judgments of their own parenting) and value-based and contextually sensitive action-taking may have not only resulted in increased parental well-being but also in more adaptive and effective parenting practices. This, in turn, may have resulted in children becoming better adjusted, as the ACT literature has suggested (Juvin et al., 2021; Leeming & Hayes, 2016; Prevedini et al., 2020; Whittingham & Coyne, 2019).

### Strengths and Limitations

Conducting this study in a natural, clinical setting was a strength. This setting gave us valuable information on how best to package and deliver the treatment so that it could meet the unique needs of the target group and ensure treatment adherence. The inclusion of novice group leaders currently undergoing training was another strength, as it allowed us to test the usefulness of the treatment manual and the effectiveness of the group leader training for therapists with limited prior knowledge of ACT. Providing clinical supervision of group leaders by the manual developers allowed us not only to monitor their skill acquisition and adherence to the manual but also allowed the manual developers to gather user feedback. At the supervision meetings and through the formal evaluations we were able to gather feedback from both treatment providers and participants at several sites, which fostered the development of the manual and enabled its developers to edit and review it before launching a RCT. One important modification that followed on the thematic analysis of parental feedback was to increase the time for parents to share experiences during the treatment sessions. Furthermore, it was a strength to have investigated transdiagnostic groups. Even if 70% of the participants were parents of autistic children, the majority of the groups also included parents of children with other disabilities. The results may speak to successful alliance-building among parents in transdiagnostic groups when there is a shared reference point—i.e., being a parent to a child with a disability. Transdiagnostic groups offer benefits such as the ability to offer treatment programs in smaller communities and rural areas and to bring together parents within a smaller radius in urban communities. Furthermore, it was a strength to have scheduled a follow-up approximately 3–4 months after the end of the intervention, as we have few studies on the long-term maintenance of effects following ACT treatment for parents (Juvin et al., 2021).

There are also several limitations to this study. First, we did not perform an Intention-to-Treat (ITT) analysis. Because it was a feasibility study, the decision was made to focus on analysis *per protocol*. In addition, it is a limitation to not have defined “other counseling” in the questionnaire, as the treatment completers had, at the time of enrollment, ongoing counseling which could have affected the outcome. We can speculate that those who had access to counseling might have been more likely to complete the program because of the additional support they received—e.g., in the form of encouragement to participate in the Navigator ACT treatment. Some of the other counseling that participants received was provided by the same disability services that organized the groups. Furthermore, even though we had a large sample size (n = 94) compared to previous studies on this topic, our comparison of completers vs. non-completers may have been underpowered. Finally, the positive outcomes must be considered preliminary, given the nature of the study (no randomization or control group). To address these limitations, we have launched a pragmatic, multicenter RCT to evaluate the efficacy of the final version of the Navigator ACT treatment.

Factors that influence treatment usefulness and attrition are other important areas for future research. This study found Navigator ACT to be a feasible and promising treatment in the context of outpatient clinical disability services, and we recommend that it undergo further evaluation within the framework of RCT.
